# Rapid emergence and transmission of virulence-associated mutations in the oral poliovirus vaccine following vaccination campaigns

**DOI:** 10.1038/s41541-023-00740-9

**Published:** 2023-09-25

**Authors:** Katharine S. Walter, Jonathan Altamirano, ChunHong Huang, Yuan J. Carrington, Frank Zhou, Jason R. Andrews, Yvonne Maldonado

**Affiliations:** 1 Division of Epidemiology, University of Utah, Salt Lake City, UT 84105 USA; 2 Department of Epidemiology and Population Health, Stanford University School of Medicine, Stanford, CA 94305 USA; 3 Department of Pediatrics, Stanford University School of Medicine, Stanford, CA USA; 4 Division of Infectious Diseases and Geographic Medicine, Stanford University School of Medicine, Stanford, CA 94305 USA

**Keywords:** Viral infection, Live attenuated vaccines

## Abstract

There is an increasing burden of circulating vaccine-derived polioviruses (cVDPVs) due to the continued use of oral poliovirus vaccine (OPV). However, the informativeness of routine OPV VP1 sequencing for the early identification of viruses carrying virulence-associated reversion mutations has not been directly evaluated in a controlled setting. We prospectively collected 15,331 stool samples to track OPV shedding from children receiving OPV and their contacts for ten weeks following an immunization campaign in Veracruz State, Mexico and sequenced VP1 genes from 358 samples. We found that OPV was genetically unstable and evolves at an approximately clocklike rate that varies across serotypes and by vaccination status. Overall, 61% (11/18) of OPV-1, 71% (34/48) OPV-2, and 96% (54/56) OPV-3 samples with available data had evidence of a reversion at the key 5’ UTR attenuating position and 28% (13/47) of OPV-1, 12% (14/117) OPV-2, and 91% (157/173) OPV-3 of Sabin-like viruses had ≥1 known reversion mutations in the VP1 gene. Our results are consistent with previous work documenting rapid reversion to virulence of OPV and underscores the need for intensive surveillance following OPV use.

## Introduction

The global use of oral poliovirus vaccine (OPV), a live, attenuated virus, has been essential in reducing the burden of poliomyelitis caused by wild poliovirus by 99.9% since 1988^1,2^. The vaccine covers all three wild poliovirus serotypes, is inexpensive, easy to deliver, and—because it is a live virus—is transmissible from vaccinated children onwards, extending the reach of vaccination campaigns. However, OPV vaccination is risky—the vaccine virus is genetically unstable and continues to evolve in vaccinated individuals and their contacts. If OPV mutates at positions responsible for attenuation of the vaccine virus, it can regain virulence. OPV can regain virulence within a vaccinated individual, causing vaccine-associated paralytic poliomyelitis, though rarely^3^. OPV may also revert to virulence over transmission chains, generating outbreaks of circulating vaccine derived polioviruses (cVDPVs).

**Fig. 1:**
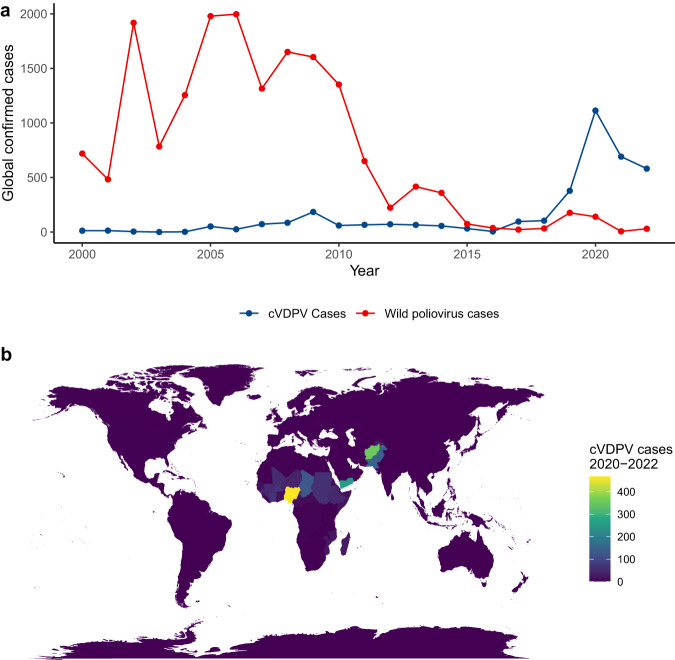
The increasing global burden of vaccine-derived poliovirus. **a** Confirmed cases of paralytic polio were reported to the World Health Organization from 2000-2022. Color indicates source: circulating vaccine derived poliovirus (cVDPV) and wild poliovirus cases. **b** Map of countries reporting cVDPV cases from 2020-2022; fill indicates number of cVDPV cases identified. Data from the World Health Organization.

To reduce the risks of cVDPVs, in May 2016, trivalent OPV vaccines (containing serotypes 1, 2, and 3) were replaced with a bivalent OPV (containing serotypes 1 and 3), following World Health Organization recommendations. The WHO recommended routine immunization with at least one dose of inactivated poliovirus vaccine (IPV) prior to the switch to bivalent OPV, to increase protection against paralytic disease^4^. However, access to IPV was limited, leaving large populations of children immunologically naïve to poliovirus type 2. Since the global switch to bivalent OPV, the majority of paralytic poliovirus cases are now caused by cVDPVs, not wild poliovirus, the majority of which are serotype 2 viruses (cVDPV2). From 2016 to 2022, type 2 cVDPVs emerged 68 times in 34 countries, leading to more than 1500 cases of paralytic polio^5^ (Fig. 1).

The GPEI recommends vaccination with OPV as part of an outbreak response to induce mucosal immunity and interrupt transmission. While in the past, monovalent OPV-2 (mOPV-2) was used to respond to cVDPV2 outbreaks, a more genetically stable replacement, type 2 novel OPV (nOPV-2)^6,7^, was recommended by the WHO under an Emergency Use Listing in 2020^8^. Since March 2021, nOPV-2 has been the primary vaccine used to respond to cVDPV2 outbreaks. Yet even with the development of a new vaccine, mOPV-2 has not been completely replaced and the risk of cVDPVs remains a serious global health concern.

Outbreak responses are guided by routine testing of cases of acute flaccid paralysis in children less 15, environmental surveillance in more than 30 high-risk countries, and sequencing of the poliovirus VP1 gene^9^. The Global Polio Eradication Initiative (GPEI) defines VDPVs by their divergence from OPV in the VP1 gene^9^ and cVDPVs as VDPVs with evidence of transmission^10,11^. The identification of new cVPDVs results in an epidemiological investigation and an immunization response^9^.

Previous genomic studies of type 2 cVDPVs and OPV-2 shedding in vaccinated children and contacts have identified strong within-host selection and tight transmission bottlenecks that govern the early evolution of type 2 viruses away from the Sabin vaccine strain along a predictable pathway to virulence^12,13^. Yet previous studies have not yet integrated detailed molecular and epidemiological data to assess the use of VP1 sequencing for the early identification of viruses posing a public health risk. Because mOPV2 as well as tOPV continue to be used in outbreak responses, we need a better understanding of the risks associated with the reversion to virulence and transmission of viruses with virulence-associated mutations, in particular, in the months directly following vaccination campaigns. Given that global surveillance and outbreak responses rely on VP1 sequencing, we need a better understanding of how informative VP1 diversity is for characterizing the risk of reversion to virulence and transmission of virulent viruses.

Here, we tracked the evolution and transmission of the three OPV serotypes during a prospective study of OPV shedding following a vaccine campaign in semi-rural communities in Veracruz State, Mexico^14^. We sequenced OPV VP1 genes from stool sampled from vaccinated children, their household contacts, and community members in unvaccinated households for 10 weeks following the vaccination campaign. This longitudinal collection provides a rare opportunity to observe the early evolution and transmission of the three OPV serotypes following vaccination campaigns and to evaluate the current use of VP1 sequencing as an epidemiological tool.

## Results

### Sample collection

**Fig. 2:**
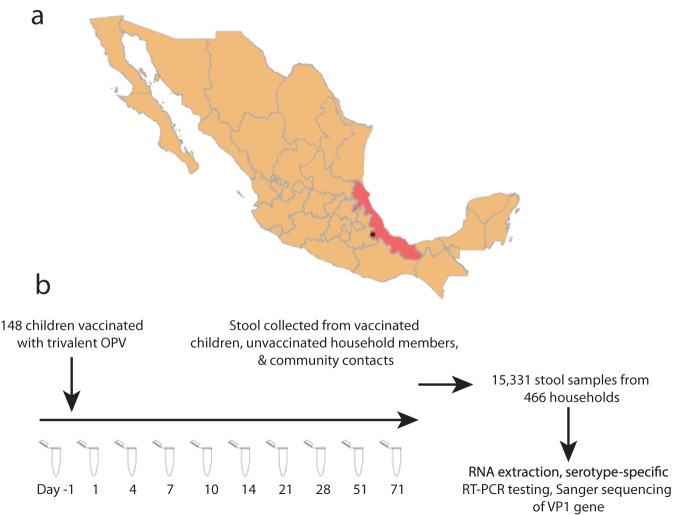
Prospective study of OPV shedding among NHW vaccinees, household members, and community members following a vaccination campaign. **a** Map of Mexico with Veracruz State highlighted and Orizaba city, where the study took place, indicated with a red point. **b** We conducted a prospective observational study of OPV viral shedding following a vaccination campaign. 148 children were enrolled and vaccinated with trivalent OPV. The stool was collected from NHW vaccinees, unvaccinated household members, and community members in unvaccinated households. We extracted RNA from stool samples, tested samples with OPV serotype-specific RT-PCR, and Sanger sequenced positive samples.

During a prospective study of OPV shedding following a National Health Week (NHW) vaccination campaign in Veracruz State, Mexico, we collected 15,331 stool samples over 10 weeks from children receiving the OPV vaccine, household contacts, and community members (Fig. 2). In our previous study of OPV shedding and transmission, we found that among 148 vaccinated children, 380 household contacts, and 1124 unvaccinated community contacts, 78%, 18%, and 7%, respectively, shed OPV^15^. We found that transmission of OPV began as soon as 1 day following OPV vaccination and, while most community members shed transmitted OPV for less than a week, we observed shedding among community members as long as 71 days after the vaccination campaign. We also observed more frequent shedding of OPV by community members in communities with the highest vaccination rates^15^.

From these samples, 551 samples were positive for at least one OPV serotype, including 267 OPV-1, 402 OPV-2, and 317 OPV-3 positive samples. We sequenced 358 high quality VP1 genes from 174 individuals, including 18.4% (49/267) positive OPV-1 samples, 33.8% (136/402) positive OPV-2 samples, and 54.5% (173/317) OPV-3 samples. Sequence data was available for 99 children who were vaccinated with OPV during the NHW vaccination campaign, whom we refer to as NHW vaccinees; and 72 community members, including 41 household contacts of NHW vaccinees. Three individuals did not have a known vaccination status. For 84 infections (unique individuals and serotypes), longitudinal samples were available at 2–5 sampling points. The prospective study was conducted in three semi-rural villages; samples include 106 individuals from Capoluca, where 70% of children less than five in participating households were vaccinated during the NHW campaign; 49 from Campo Grande, where 30% were vaccinated; and 19 individuals from Tuxpanguillo, where 10% were vaccinated.

### Genetic instability of OPV

**Fig. 3:**
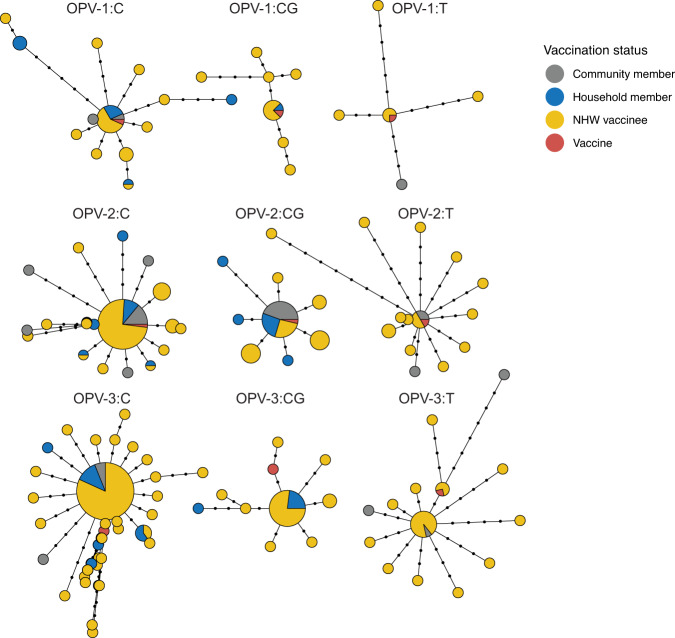
Limited overall genetic diversity in sampled OPV viral capsid protein 1 (VP1) gene following vaccination campaigns. For each OPV serotype and sampling site, haplotype networks of OPV VP1 represent sampled genetic diversity. Nodes indicate identical haplotypes (VP1 consensus sequences) and node size indicates the number of samples sharing a haplotype. Points on edges between nodes indicate the SNP distance between haplotypes and node colors indicate an individual’s vaccination status. Labels indicate OPV serotype and study site. Site C Capoluca, had 70% vaccination coverage of eligible children, site CG Campo Grande: 30%, site T Tuxpanguillo: 10%. One sample with a distant haplotype (>14 SNPs distant to the Sabin vaccine) was removed from OPV-2 site C, to aid visualization.

We observed a rapid rise of VP1 diversity in all three serotypes over weeks following OPV vaccination. The most common haplotype for both OPV-1 and OPV-2 in each sampling location represents the Sabin vaccine strain (Fig. 3) and we observed a star-like pattern in the haplotype networks, where most haplotypes were directly connected to the Sabin haplotype. Outside of the dominant haplotype, most haplotypes are represented by a single sample.

In contrast, for OPV-3, the dominant haplotype differs by a single nucleotide polymorphism (SNP) from the vaccine strain, a previously described attenuating site, C2493U (Fig. 3). Again, there is a star-like pattern of OPV-3 diversity, with many branches stemming directly from the dominant haplotype. As with OPV-2 and OPV-3, most haplotypes outside the dominant haplotype are represented by a single sample.

### Molecular evolution of OPV following vaccination campaigns

**Fig. 4:**
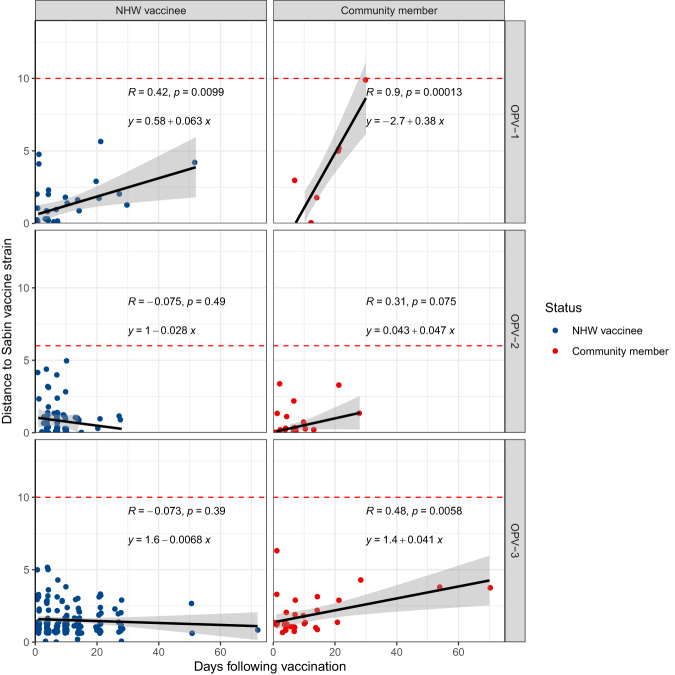
Measurable evolution in the OPV VP1 gene following vaccination campaigns. Genetic distance of the OPV VP1 gene to the Sabin vaccine strain versus time from vaccination for NHW vaccinees (blue) and not vaccinated study participants, including household contacts and unvaccinated community members (red), for each OPV serotype (a-c). Black lines and grey shading of a linear model for distance to the Sabin strain. Days following vaccination is measured as days from the first vaccination (for NHW vaccinees), day from the first household vaccination (for household members), and days from the first community vaccination (for other community members). Red dashed lines indicate the genetic distance threshold for a VDPV: 10 SNPs distant from the Sabin vaccine for serotypes 1 and 3 and 6 SNPs distant for serotype 2.

The Global Polio Eradication Initiative (GPEI) defines VDPVs by their divergence from the Sabin vaccine in the VP1 gene (>1% divergent or ≥10 substitutions for serotypes 1 and 3, 0.6% divergent of ≥6 substitutions for serotype 2)^9^. The majority, 99% (338/340), of all samples sequenced following vaccination fell under the thresholds for a VDPV. However, one sample from an NHW vaccinee, collected 4 days after vaccination met the definition of a VDPV2 (VP1 genetic distance 14 from Sabin 2), and one sample from a household contact sampled 30 days following vaccination met the definition of a VDPV1 (10 SNPs distant from Sabin 1) (Fig. 4).

We then tested whether OPV accumulated substitutions in a clock-like and, therefore, if OPV divergence from Sabin vaccine strains could be used to estimate the duration of viral circulation in the months following a vaccination campaign. We were not able to calculate a true molecular clock rate because of the limited observation period and because of our inclusion of sequences that do not represent a single genetic lineage. Genetic divergence of OPV-1 was significantly associated with time following vaccination in both NHW vaccinees (*p* = 0.0099, Pearson’s r = 0.42) and community members (*p* < 0.001, Pearson’s r = 0.90) (Fig. 4). When considering all samples together, the observed mutations in OPV-1 corresponded to a VP1 gene evolutionary rate of 1.2 × 10^-4^ substitutions per site per day. Genetic divergence of OPV-2 was not significantly associated with time following vaccination in NHW vaccinees (*p* = 0.49, Pearson’s r = −0.075) and was positively correlated with time among community members, though not significantly so (*p* = 0.075, Pearson’s r = 0.31) (Fig. 4). OPV-3 divergence was positively associated with time following vaccination for community members (*p* = 0.0058, Pearson’s r = 0.48), but not NHW vaccinees (*p* = 0.39, Pearson’s r = −0.073)

We reasoned that mutations at known attenuating positions may be under strong selection and could affect an estimate of the molecular clock. However, excluding the known attenuating sites in the VP1 gene did not substantially change observed associations between genetic divergence and time (Supplementary Fig. 1).

### Moderate signal of recent transmission in OPV VP1 gene sequence diversity

VP1 genetic diversity is also used by the GPEI to determine if a VDPV is genetically linked to previously collected environmental or clinical samples, which makes it a cVDPV, and whether viruses are linked to a previously identified cVDPV emergence or constitute a new emergence.

**Fig. 5:**
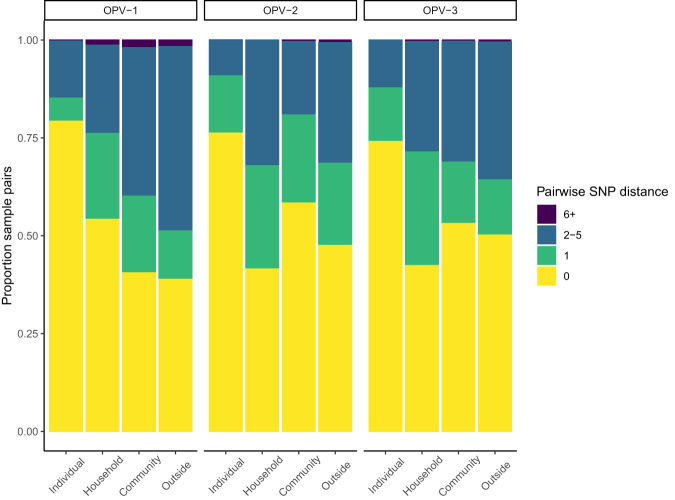
Moderate genetic structure of OPV VP1 gene from samples shed two months following vaccination campaigns. The proportion of pairs of OPV VP1 sequences within a given, binned pairwise genetic distance for pairs of samples collected longitudinally from the same individual, from the same household, different households within the same community, or outside the community, i.e. pairs of samples from different communities. Facets indicate OPV serotype.

To evaluate the genetic structure of VP1, we compared pairwise genetic distances across samples. As expected with the relatively short duration of sampling following vaccination campaigns, 27.9% (644/2304) of OPV-1; 41.9% (6693/16,616) of OPV-2; and 41.4% (12,272/29,628) of OPV-3 VP1 pairs of sequences were identical. The mean pairwise distance was 2.5 SNPs for OPV-1, 1.4 SNPs for OPV-2, and 1.3 SNPs for OPV-3 (Fig. 5).

In a general linear model, the pairwise genetic distance between VP1 samples was elevated for individuals from the same household (aOR: 1.31; 95% CI: 1.12–1.52), from the same community (aOR: 1.38; 95% CI: 1.26–1.53), and from different communities (aOR: 1.53; 95% CI: 1.39-1.769), compared to samples collected from an individual. As expected, longitudinal samples collected from the same individual were largely identical. Pairwise genetic distances differed across serotypes: compared to OPV-1, we observed decreased pairwise genetic distances for both OPV-2 (aOR: 0.59; 95% CI: 0.57–0.61) and OPV-3 (aOR: 0.54; 95% CI: 0.52–0.55).

### Rapid loss of OPV attenuating mutations following vaccination campaigns

We then examined known mutations at key attenuating sites in the vaccine virus, associated with the reversion of vaccine viruses to virulence. First, as stool may contain viruses with both revertant and non-revertant alleles at the same locus, we assessed the proportion of viral RNA in each sample with a revertant mutation at the major attenuating position in the 5’ UTR, or the revertant proportion, with an allele-specific qPCR test^16^. Of the 122 samples with available results, 99 (81%) had a signal of reversion to virulence (revertant proportion > 0), and in 82 samples (67%), the reversion proportion was ≥ 50%. Among samples with complete assay information, we found evidence of a 5’ UTR reversion in 82% (87/106) of vaccines, 85% of household contacts (11/13), and 33% (1/3) of other community members. We identified transmission of the 5’ UTR reversion mutation in six independent households in which the revertant mutation was first identified in NHW vaccinees and subsequently identified in household members (4 households) or concurrently identified in both NHW vaccinees and household members (2 households). Transmission of the 5’ UTR reversion mutation occurred for all three serotypes.

**Fig. 6:**
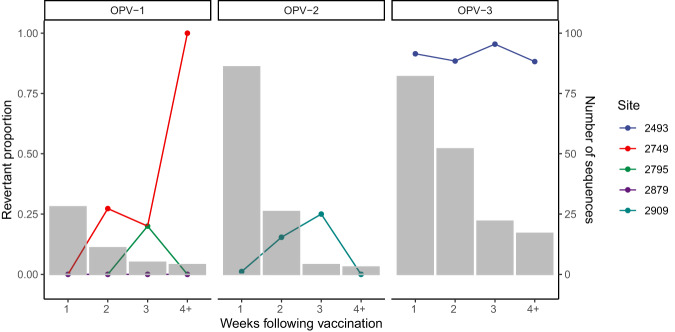
Loss of OPV attenuating mutations following vaccination. For each serotype, points indicate the revertant proportion, the proportion of samples with a mutation at a key attenuating position in OPV in the weeks following vaccination. Bars indicate the total number of sequences available for that sampling week. Each serotype has unique attenuating sites.

We then examined additional mutations within the VP1 gene associated with reversion to virulence. Of three known virulence attenuating substitutions in the OPV-1 VP1 gene, 16% (8/49) of samples had a A2749G (Sabin to virulent wildtype) mutation, including 4 NHW vaccinees and 2 household contacts. The mutation first appeared 14 days following vaccination in a vaccinated child and contact in one house and in another vaccinated child independently and rose in frequency, although there were limited OPV-1 samples available 4+ weeks after vaccination. It was observed in stool up to 52 days after the vaccination campaign. Three samples with the A2749G mutation, including two NHW vaccinees and one household member, also had evidence of fixation of the reversion mutation in the 5’ UTR (reversion proportion of 100%). A single sample shed by a vaccinated child harbored a A2795G substitution, and no U2879C attenuating mutations occurred (Fig. 6).

Among OPV-2 samples, the known VP1 reversion mutation, U2909C/A, occurred in 4.4% (6/136) of samples, in four NHW vaccinees. The substitution was first observed 7 to 14 days after vaccination; two children shed OPV-2 carrying this reversion mutation on multiple days. The mutation was observed in stool up to 21 days following the vaccination campaign, though again, there were limited OPV-2 samples available 4+ weeks after vaccination. No individuals had mutations present at the adjacent site 2908, also associated with reversion^7^. Four of these samples also had evidence of a reversion mutation in the 5’ UTR.

Among OPV-3 samples, the majority, 96.5% (167/173), had a C2493U eversion substitution and an additional 11 samples had a mixed allele or no allele call at that site, possibly evidence of mixed infection. Samples with a C2493/U mutation included 95% (89 of 94) of individuals with OPV-3 sequences available, including 62 NHW vaccinees, 19 household members, and 8 individuals outside of vaccinated households. The mutation first appeared one day following the vaccination in NHW vaccinees, household members, and individuals outside vaccinated households and it was observed in stool up to 72 days after the vaccination campaign from a vaccinated child and 70 and 54 days from virus shed in a household member and community member, respectively. 35% (58/167) of these samples also had evidence of a reversion mutation in the 5’ UTR.

## Discussion

While rapid reversion to virulence has been documented in OPV, the risk of transmission of OPV with virulence-associated mutations following vaccination campaigns is less clear. Further, global poliovirus surveillance uses molecular epidemiology to directly guide outbreak responses. However, the informativeness of the routinely sequenced marker gene, VP1, in the early identification of VDPVs posing a public health risk has not been evaluated in a controlled setting.

Here, we leveraged OPV sampled for 10 weeks following an immunization campaign in a population previously vaccinated with IPV to assess the epidemiological information within VP1 samples. We found that all three OPV serotypes were genetically unstable in the weeks following vaccination campaigns, consistent with previous reports. We identified a linear relationship between genetic divergence from vaccine strains and sampling time (though this signal differed by vaccine status) and significant genetic structure. Consistent with prior reports, we found evidence of frequent reversion substitutions among children who had received the OPV vaccine. Importantly, reversion mutations were frequently also found among community members who had not received OPV as part of the National Immunization Week, evidence that revertant viruses may be frequently transmitted onwards.

Polioviruses are among the fastest evolving RNA viruses, and we observed that the rapid generation of genetic diversity in NHW vaccinees and their household and community contacts contains epidemiological information. Robust molecular clock estimates require samples from a well-defined virus lineage and over a significant sampling period, which were not available here, and therefore, we report trends observed in the genetic divergence from the Sabin vaccine over time rather than precise estimates of the OPV molecular clock. We found that the early evolution of OPV is largely clocklike, with variation in mutation rate across serotypes. The lack of a signal of clocklike evolution of OPV-2 and OPV-3 in NHW vaccinees likely reflects the shorter sampling period for NHW vaccinees; unvaccinated household and community contacts were infected later in the study by viruses that had already passed through at least one host.

Though we were not able to calculate a true molecular clock rate, our observed OPV-1 VP1 substitution rate of 1.2 × 10^−4^ substitutions/site/day (including all samples) was similar to previous estimates of OPV-1 evolution 0.83 × 10^−4^ substitutions/site/day^17^ (estimated from three years of surveillance for acute flaccid paralysis). Previously, the molecular clock for wild poliovirus type 1 was estimated to be 0.28 × 10^−4^ substitutions/site/day, from a set of 31 viruses sampled over ten years^18^ 3.12 × 10^−5^ substitutions/site/day in samples shed from a chronically infected immune-compromised individual^19^. The differences in substitution rate may reflect the different sampling durations, as shorter sampling durations often yield higher pathogen substitution rate estimates, likely because purifying selection has not yet removed transient deleterious mutations that are still present prior to the effect of purifying selection^20,21^, in addition to different selective pressures in these different sampling contexts.

We observed two samples with unexpectedly high levels of genetic divergence that met the definition of VDPVs. Likely, these samples represent OPV infection from sources outside of the focal NHW vaccination campaign, indicating prolonged replication or circulation elsewhere.

We observe moderate genetic structure in VP1 sequences, with increasing genetic divergence between samples from different communities compared to that sampled within the same community, household, or longitudinally within an individual. Whole genome sequences and additional epidemiological information about the timing of infection and contacts could improve the inference of transmission networks.

The rapid reversion to virulence of Sabin vaccines is well documented^22,23^. While each serotype has key attenuating mutations in the 5’ UTR, additional mutations along the genome, including in the VP1 gene, stabilize the attenuated phenotype. In OPV-2, a predictable evolutionary route towards virulence includes gatekeeper mutations in the 5’UTR and the VP1 gene, followed by recombination events, followed by additional stabilizing mutations^12,13^. Most samples had evidence of the major 5’ UTR reversion mutation, including samples 82% of vaccinee samples, 85% from household contacts, and 33% from other community members. Importantly, we also found evidence of household transmission of the 5’ UTR mutation for all three serotypes, indicating that samples that do not meet the genetic distance threshold for a VDPV are both virulent and transmissible.

We additionally found a high frequency of attenuating substitutions in VP1, with substitutions appearing early after vaccination. The higher frequency of OPV-2 and OPV-3 reversion mutations we observed could reflect the greater observed genetic stability of OPV-1, which differs from the virulent wild-type progenitor virus by 59 substitutions, compared to OPV-2 and OPV-3 which are more closely related to the virulent ancestor^24^. Our group previously found that 97% of OPV-3 recipients shed viruses that had a site 472 reversion mutation within two weeks following vaccination^25,26^. The instability of OPV-3 has epidemiological consequences: OPV-3 causes the majority of vaccine-associated paralytic poliomyelitis (VAPP), paralysis attributable to the vaccine itself, followed by OPV-2 and then OPV-1^3^. However, previous studies have reported heterogeneity at the OPV-3 C2493U attenuating mutation in vaccine stocks^27^. We did not have access to vaccine stocks and therefore cannot determine if the near-fixation of an attenuating mutation we observed was due to heterogeneity in the vaccine or de novo evolution in NHW vaccinees and subsequent onward transmission.

OPV remains the primary tool used to control poliovirus outbreaks. Novel OPV2 (nOPV-2), a genetically stable replacement for mOPV-2, was granted Emergency Use Listing in 2020 and first used in outbreak vaccination responses in 2021^8^. Since then, over 600 million doses of nOPV2 have been administered and the WHO Strategic Advisory Group of Experts on Immunization recommended the transition from its initial use period to wider use because of its documented safety and effectiveness in inducing strong immune responses in a cVDPV2 outbreak response^8,28,29^. The increasing replacement of mOPV-2 is expected to reduce the risk of cVDPVs in the future; however, several questions remain about how nOPV-2 can be optimally used for outbreak control^30^.

Although the majority of OPV samples in our study collected were defined as Sabin-like viruses, rather than VDPVs, many of the Sabin-like viruses had rapidly lost attenuating mutations at previously described positions. While VP1 sequence divergence may be useful in estimating the duration of circulation or predicting transmission clusters, circulating OPV that does not yet meet GPEI definitions for a cVDPV, can be virulent and can pose a public health risk in populations without prior immunity. For example, Sabin-like OPV-2 viruses, which were 3 SNPs distant to the Sabin virus VP1 sequence, were previously found to have caused a cluster of acute flaccid paralysis cases in an orphanage in the Altai Region of Russia^31^. All outbreak genomes shared 15 substitutions compared to the Sabin virus, including the loss of attenuation mutations A481G and U2909C.

The global increase of cVDPV2 outbreaks has increased dramatically since 2016, when Sabin 2 was removed from the trivalent poliovirus vaccine^5^. Our results are consistent with previous work on the genetic instability of OPV and rapid reversion to virulence and underscore the urgency of heightened surveillance during and following vaccination campaigns.

## Methods

### Prospective sampling

We conducted a prospective study of OPV shedding and transmission following a trivalent OPV vaccination campaign during Mexico’s February 2015 National Health Week (NHW) in three semi-rural populations in Orizaba, Veracruz State^14^ (Fig. 2). Wild poliovirus has not been reported since 1994 in Mexico. Mexico’s national vaccine program has included routine IPV as well as two annual OPV vaccination campaigns since 2007 and therefore provides a model for transmission of OPV in a setting with routine IPV immunization, an epidemiological context that will increasingly exist as IPV replaces OPV and OPV is only used as a tool for cVDPV or wild poliovirus outbreak response.

We randomized 466 households in three communities to the vaccine and no-vaccine arm of our study. To measure the impact of vaccination rate on the frequency of household and community shedding, we randomized households so that community-level OPV vaccination rates were 10%, 30%, and 70% in the three communities^14^. In total, 148 children (8.5% of the population) were vaccinated with trivalent OPV during the NHW. 148 children receiving the vaccine during the NHW (NHW vaccinees), 380 household contacts, and 1124 community members from households that did not receive OPV during the NHW were followed prospectively and stool was collected from all members of sampled households 1 day prior to the National Health Week OPV immunization campaign, and 1, 4, 7, 10, 14, 21, 28, 51, and 71 days following, for a total of 10 samples per person^14^.

Study nurses collected immunization histories and additional demographic and clinical metadata from all study participants. We report rates of OPV shedding and transmission among child vaccines, household contacts, and community contacts in prior studies^15^; the focus of this analysis was on the molecular evolution of tOPV following vaccination campaigns.

The study was approved by the Stanford University Institutional Review Board (Protocol #31546), the Comité de Etica, Bioseguridad e Investigación of the Instituto Nacional de Salud Pública (CI: 1260, No. 1581), and the Instituto Veracruzano para la Formación e Investigación en Salud (SESVER/IVEFIS//SIS/DIB/0109/02014, classification 15 S).

### Sanger sequencing of VP1 gene

We extracted RNA from frozen stool samples using an RNeasy minikit (Qiagen, Valencia, CA) according to the manufacturer’s protocol. Positive and negative controls were included in each set of extractions. Extracted RNA preparations were stored at −80°C and tested samples for OPV with serotype-specific RT-qPCR^14,16^. We Sanger sequenced the VP1 gene using the Y7 and Q8R primers^32^ and generated consensus sequences by aligning forward and reverse sequences to serotype-specific reference genomes in *Geneious* v7.

### 5’ UTR revertant proportion

We additionally used a previously described allele-specific RT-qPCR assay to assess the proportion of viral RNA in each sample with the major 5’ UTR reversion mutation, or the revertant proportion^16^. We combined five microliters of the reverse transcription reaction mixture with 10 μl TaqMan universal PCR master mix without uracil-N-glycosylase (Applied Biosystems, Foster City, CA), 0.05 μl TaqMan probe (Applied Biosystems, Foster City, CA), 0.9 μl each of forward and reverse primers, and 3.15 μl distilled water. Cycling conditions were as described previously^25^. Samples that returned average cycle threshold (CT) values of >35 from duplicate assays were considered negative, due to an increased number of false-positive results above this threshold.

We calculated the revertant proportion from the allele-specific RT-qPCR cycle threshold values as previously described^16^: 2^−rev*CT*^/(2^−rev*CT*^ + 2^−nonrev*CT*^), where rev*CT* and nonrev*CT* are revertant and nonrevertant *C*_*T*_ values, respectively.

### Bioinformatic analysis

We aligned VP1 gene sequences of each OPV serotype to the corresponding Sabin vaccine sequence (GenBank Accession Numbers: AY184219, AY184220, AY184221) with MAFFT v.7^33^, with the algorithm recommended for closely related viral sequences (https://mafft.cbrc.jp/alignment/software/closelyrelatedviralgenomes.html), preserving the alignment length of the reference sequence. We used the default alignment strategy and a nucleotide sequence scoring matrix of 1PAM / κ = 2, recommended for closely related DNA sequences.

We quantified pairwise distances between samples with the R package *ape* (pairwise deletion = TRUE)^34^. For household contacts and community members who were not vaccinated with OPV, we measured the time since vaccination as the time since vaccination of the household member or the earliest vaccination given in the community, respectively. We visualized haplotype networks built from minimum spanning trees with the R package *pegas*^35^.

### Reporting summary

Further information on research design is available in the Nature Research Reporting Summary linked to this article.

### Supplementary information

Supplementary Information

Reporting Summary

## Data Availability

The full-length VP1 sequences used in our study are publicly available on GenBank (accession numbers OQ714877 - OQ715012, OQ715013 - OQ715185, OQ715186 - OQ715234). The references sequences used for sequence alignment are publicly available on GenBank (AY184219, AY184220, AY184221). Multiple sequence alignments and metadata to reproduce figures and analyses are publicly available on GitHub (https://github.com/ksw9/opv-consensus). We accessed World Health Organization poliovirus case data from the WHO extranet (https://extranet.who.int/polis/public/CaseCount.aspx), including both global and country-level reported cases from 2000–2022.

## Code availability

Code for analysis and figures is publicly available on GitHub (https://github.com/ksw9/opv-consensus).
