# Supplementary material for: Rapid emergence and transmission of virulence-associated mutations in the oral poliovirus vaccine following vaccination campaigns
Source: NPJ Vaccines. 2023 Sep 25;8:137. doi: 10.1038/s41541-023-00740-9 (PMC10520055; doi:10.1038/s41541-023-00740-9)

Supplementary Information

Supplementary Figure 1. Molecular evolution in the OPV VP1 gene following vaccination campaigns when excluding attenuating mutations. Genetic distance of the OPV VP1 gene to the Sabin vaccine strain versus time from vaccination for NHW vaccinees (blue) and not vaccinated study participants (red), for each OPV serotype (a-c). Black lines and grey shading of a linear model for distance to the Sabin strain. Days following vaccination is measured as days from first vaccination (for NHW vaccinees), day from first household vaccination (for household members), and days from first community vaccination (for other community members). Red dashed lines indicate the genetic distance threshold for a VDPV: 10 SNPs distant from the Sabin vaccine for serotypes 1 and 3 and 6 SNPs distant for serotype 2.

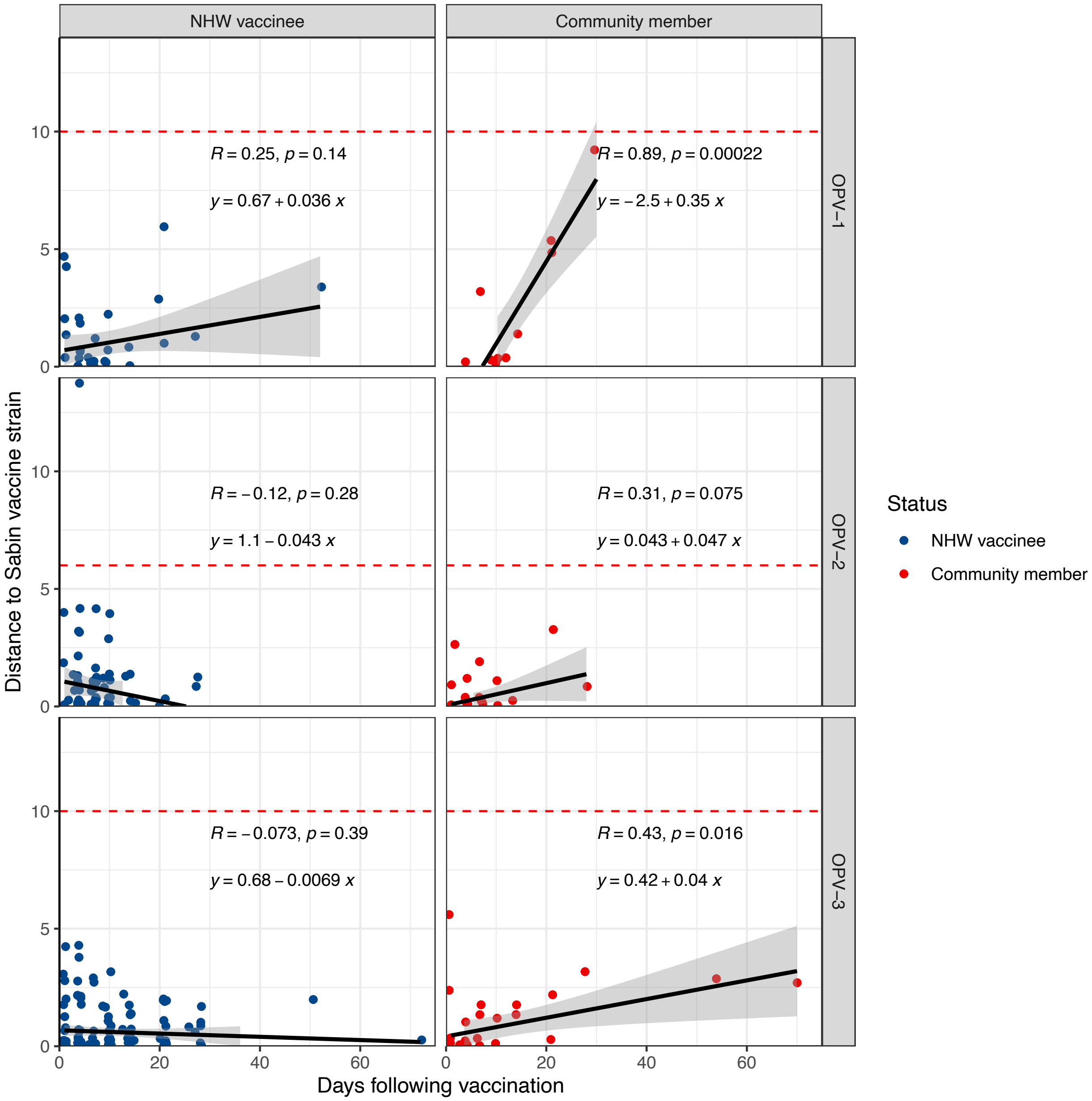

Supplement: Supplementary file 1 — Supplementary Information [file 41541_2023_740_MOESM1_ESM.pdf]
